# Primary Hyperparathyroidism: 11-Year Experience in a Single Institute in Thailand

**DOI:** 10.1155/2012/952426

**Published:** 2012-05-31

**Authors:** Poramaporn Prasarttong-Osoth, Pakpong Wathanaoran, Waraporn Imruetaicharoenchoke, Supakorn Rojananin

**Affiliations:** Department of Surgery, Faculty of Medicine, Siriraj Hospital, Mahidol University, Bangkok 10700, Thailand

## Abstract

Primary hyperparathyroidism (PHPT) is not an uncommon disease in the western countries. In Thailand, on the contrary, PHPT was a rare condition with various clinical presentations. All 45 PHPT patients who underwent parathyroidectomy at the Department of Surgery, Siriraj Hospital during January 1997 and December 2007 were retrospectively reviewed. Demographic data, clinical presentation, localizations imaging, operative procedures, findings, complications, and pathological reports were analyzed. Median age was 49 years (range 15–89 years) with female: male ratio of 3 : 1. Only one patient (2.2%) was asymptomatic PHPT. Of all symptomatic cases, 30 (66.7%) had skeletal symptoms, 7 (15.6%) had renal impairment, and 39 (86.7%) had mixed symptoms. 42 patients (93.3%) had parathyroid scan and all had bilateral exploration of the neck. Postoperative hungry bone syndrome was noted in 10 patients (22%). On followup, skeletal and neuropsychiatric symptoms were improved but the renal impairment was remained. The *s* small number of asymptomatic PHPT in our study may refer to large number of underdiagnosed PHPT in general population. The guideline for screening serum calcium for diagnosis of PHPT in Thai populations will improve the long-term consequence of the disease but will need further information to identify the target group.

## 1. Introduction

Primary hyperparathyroidism (PHPT) is a condition caused by excessive and uncontrolled secretion of parathyroid hormone by the abnormal parathyroid gland(s). In the west, this condition is common between the fifth to seventh decades of life with female : male ratio of 3 : 1. Incidence of PHPT was estimated to be 20 per 100,000 [1, 2] and more than 80% of PHPT cases is asymptomatic at the time of diagnosis [3–5]. The first sign of PHPT is elevated serum calcium on a routine biochemical evaluation.

On the contrary, PHPT is relatively rare disease in Thailand. There was no previous published data regarding PHPT in Thai populations. Most of PHPT patients in Thailand presented with multiple somatic signs and symptoms of many systems including skeletal system (bone pain, osteitis fibrosa cystica, pathological fracture, and proximal muscle weakness), renal system (recurrent nephrolithiasis, renal failure), nervous system (psychosis, mental state change), GI system (peptic ulcer, constipation, and pancreatitis), and hypertension. 

Understanding the clinical presentation and its course of PHPT in Thai populations would benefit in future detection and management of PHPT in Thailand. In addition, we can compare these information to other Asian countries where screening of hypercalcemia is not the general practice.

## 2. Materials and Methods

A retrospective descriptive study of patients with PHPT who underwent parathyroidectomy at Department of Surgery, Siriraj Hospital during January 1997 and December 2007 was carried out. Patients with recurrent primary hyperparathyroidism, secondary and tertiary hyperparathyroidism were excluded. The study was approved by the Institutional Review Board with the exemption of informed consent. 

All related medical data including demographic data, clinical presentations, comorbidities, localizations of abnormal parathyroid glands, operations and their findings, and pathology and complications were reviewed. Demographic data consisted of age, sex, and other diagnosis. Clinical presentations were divided into different physiological systems as skeletal, renal, neuromuscular, neuropsychiatric, gastrointestinal systems, and other nonspecifics symptoms. Data on blood chemistry included preoperative and postoperative serum calcium (total and ionized), phosphate, alkaline phosphatase, creatinine, and parathyroid hormone. Postoperative blood chemistry was collected at 3–12 months. Imaging modalities for localization and their accuracy when comparing to operative findings were noted. 

All data were prepared and analyzed statistically using the Statistical Package for the Social Sciences program version 13.0 for Windows (SPSS Inc, Chicago, IL, USA). Categorical data were calculated as percentages and were analyzed.

## 3. Results

45 new cases of PHPT underwent parathyroidectomy at Siriraj Hospital between January 1997 and December 2007. The age ranged from 15 to 89 years (Average 49 years) with female: male ratio of 3 : 1. Disease prevalence was distributed in all age group as shown in Table 1. Mean follow-up period was 3 years (range 6 months to 7 years). There was no mortality in this study. 

In this study, there was only 1 asymptomatic patient (2.2%) at the time of diagnosis. The majority of 44 patients (97.8%) were presented with various symptoms. In this group 39 patients (86.7%) had mixed multisystem symptoms. The most common presentation was skeletal symptoms (30 patients, 66.7%) with one-fifth of all patients had pathological fracture. The second most common clinical features were in renal system. In 21 patients (46.7%) who had renal symptoms, 7 patients (15.6%) had renal function impairment. The only one patient who presented with a neck mass was one of four of parathyroid carcinoma. Interestingly, 2 patients were diagnosed during postpartum period due to fetal hypocalcemia. Details of clinical presenting symptoms were shown in Table 2. 

 In this study, median serum calcium at the time of diagnosis was 13.1 mg/dL (range 8.1–22). Median parathyroid hormone level was 341 pg/mL (range 107–2490 pg/mL). It was noted that the highest level of serum parathyroid hormone detected was 2490 pg/mL and was found in patient with parathyroid adenoma not carcinoma. Elevated alkaline phosphatase was documented in 36 patients (80%) with the median of 453 unit/L (range 60–3 unit/L). As in this study there was a high proportion of parathyroid cancer, we also presented the figures of blood chemistry of all cases and cases excluding carcinoma to be compared (Table 3). 

 Preoperative localization with the parathyroid scan (Tc-99 m pertechnetate/Tc-99 m MIBI subtraction and dual phase Tc-99 m MIBI and SPECT) was selected as a primary investigation in 42 patients (93.3%). The parathyroid scan yielded true positive scan in 39 patients (92.9%). Of three patients who did not have parathyroid scan, two had ultrasound scan and one had no preoperative imaging for localization. One patient who had negative parathyroid scan had disease gland localized by MRI scan. 

 All patients had standard collar incision with bilateral neck exploration. The abnormal parathyroid gland can be identified in 97.8% (44 patients). Median sternotomy was performed due to location of the disease gland in the mediastinum in one patient. Pathological examination revealed single adenoma in 79.5% (*n* = 35), hyperplasia 11% (*n* = 5), and parathyroid carcinoma 9% (*n* = 4). 

 On the postoperative course, 10 patients (22%) had severe and prolonged hypocalcemia, known as hungry bone syndrome. This condition was well related to preoperatively high level of alkaline phosphatase (median 606 unit/L, range 320–3384 unit/L). In addition, hungry bone syndrome was also found in patients who had severe skeletal symptoms and signs. There was no report of recurrent laryngeal nerve injury, postoperative bleeding, and wound infection. One patient had thoracic duct injury and was treated successfully without surgical correction. 

All patients with pathological fracture (*n* = 9) had significant clinical improvement demonstrated by well union of the fracture sites within 5–20 months postoperatively. One patient who had preoperative psychosis was gradually improved. However, patients with nephrocalcinosis did not show any improvement of the kidney. Most of the patients with preoperative renal failure (serum creatinine > 2) also did not get benefit on the renal function after parathyroidectomy.

## 4. Discussion

PHPT was traditionally described with classical symptoms and signs as painful bones, renal stones, abdominal groans, and psychic moan [6]. However, after measurement of serum calcium levels was included in the routine check-up program in many developed countries, number of asymptomatic cases has been increasing, therefore symptomatic PTHT has become the minority. 

In many Asian countries, routine testing of serum calcium is still not in a general practice. Clinical reports from India and Taiwan show all higher percentage of symptomatic PHPT compared to the West [7–9]. In Thailand serum calcium monitoring is also not a routinely practice, therefore our study documented only 2.2% of asymptomatic presentation in PHPT. The real incidence of PHPT in Thai population is unknown. However, we believed that PTHT is definitely underdiagnosed and delay detected in Thailand. 

Age distribution of PHPT in our study seems to expand in every age group (15–89 years) which is similar to India but not Taiwan. To design a screening age group of PHPT for early detection in Thailand would need further information. 

Skeletal-and-renal related symptoms were the leading clinical presentation of symptomatic PHPT. In this study there were 20% of patients with pathological fracture and 15.6% with renal impairment. These clinical features represented the late diagnosis and showed similar pattern of symptoms to other Asian countries. Although all fractures were reunioned after parathyroidectomy, the period of treatment was relatively longer than in healthy bone (5–20 months). Anyhow, the renal consequences such as renal calculi and impairment may not improve after parathyroidectomy. 

Interestingly, on analysis without patients with parathyroid cancer, the blood chemistry on diagnosis showed no significant different. These may also refer that late diagnosis of PHPT could mimic the clinical pictures of parathyroid carcinoma. 

Other findings of high number of symptomatic cases lead us to quest that there may be many more hidden asymptomatic cases in Thai population. However, the answer will fall into two situations. First, asymptomatic cases in Thai were distributed in all age group, this situation made screening protocol difficult due to varied target group and low cost-effectiveness. The second situation, if asymptomatic cases in Thai were clustered in age group 55–75 years. The next question was that do screening and parathyroidectomy in those patients really reduced consequences of PHPT, as some of the symptomatic cases were young and will be excluded in screening protocol. However, authors believed that in unknown situation, screening serum calcium in female age >55 or menopausal age group would gain some benefits. Thus, we encourage addition of serum calcium screening for female age >55 who will have routine blood check up for other purposes. 

Localization method with parathyroid scan had been reported with accuracy 50–90% [10]. In this study, 93.3% of cases were localized with parathyroid scan with accuracy of 92.9%. Bilateral exploration was still the operation of choice due to not very high volume of PHPT in our experience. With bilateral exploration, there was still one case where the abnormal parathyroid gland could not be identified. This patient was also the one who had negative preoperative imaging localization. 

Finally, pathological findings of the abnormal parathyroid gland in this study were revealed single adenoma in 79.5% (*n* = 35), hyperplasia 11% (*n* = 5), and parathyroid carcinoma 9% (*n* = 4). This high proportion of parathyroid cancer may lead us to think of possibility of many undiagnosed benign cases in the population. In Taiwan study, with screening of serum calcium in 4359 healthy individuals aged 21–94 years, at least 4 cases were diagnosed with PHPT, representing a minimum estimated prevalence of 92 per 100,000. This figure is not very far from report from the western countries.

## 5. Conclusion

Our experience with PHPT from a single institute in Thailand was likely the late diagnosis PHPT with various severe symptoms. Anyway to improve our detection will definitely benefit the patient in duration of treatment and prevention of permanent damage to major organs. Clinical features of late diagnosis of benign PHPT may also mimic the presentation of parathyroid carcinoma. According to our findings, there might be the stage of underdetection of asymptomatic hypercalcemia. The guideline for screening serum calcium in order to diagnose PHPT in Thai populations would need further information to develop.

## Figures and Tables

**Table 1:** Characteristic of patients by age group and gender.

Age group (years)	Female	Male
<30	5	1
31–40	5	5
41–50	8	1
51–60	6	2
61–70	6	1
>71	4	1

Total	34	11

**Table 2:** Details of symptoms in according to system.

Symptoms	Percentage (*n*)
*Asymptomatic hypercalcemia*	2.2 (1)
Skeletal symptoms	
Pathological fracture	20 (9)
Radiological changes	31.1 (14)
Osteoporosis	33.3 (15)
Chondrocalcinosis	2.2 (1)

Total	66.7 (30)

Renal symptoms	
Renal calculi, ureteric calculi	28.9 (13)
Renal insufficiency (Cr > 2)	15.6 (7)
Polyuria	11.1 (5)

Total	46.7 (21)

Neuromuscular symptoms	
Muscle weakness	17.8 (8)
Myalgia	24.4 (11)
Peripheral neuropathy	2.2 (1)

Total	44.4 (20)

Neuropsychiatric symptoms	
Alteration of consciousness	8.9 (4)
Psychosis	2.2 (1)

Total	11.1 (5)

Other symptoms	
Weight loss	11.1 (5)
Abdominal pain	4.4 (2)
Peptic ulcer	8.9 (4)
Constipation	11.1 (5)
Chronic pancreatitis	2.2 (1)
Neck mass	2.2 (1)
Fetal hypocalcemia	4.4 (2)
Anorexia	2.2 (1)

Total	36.7 (17)

**Table 3:** Blood chemistry in pre- and postoperative status.

Parameters	Preoperative (45 patients)	Preoperative (41 patients, excluded parathyroid carcinoma)	Postoperative (45 patients)	Postoperative (41 patients, excluded parathyroid carcinoma)
Total calcium (8.1–10.4 mg/dL)	13.1 (8.1–22)	13.1 (8.1–22)	9.15 (8–13)	9.19 (8–11)
Ionized calcium (4.6–5.2 mg/dL)	6.2 (3.8–10.8)	6.2 (3.8–10.8)	4.5 (3.6–8.9)	4.7 (3.6–8.9)
Phosphate (2.2–5 mg/dL)	2.4 (1.2–7.9)	2.4 (1.2–7.9)	2.9 (1.5–4.6)	3.7 (1.5–2.3)
PTH (15–65 pg/mL)	341 (107–2490)	330.5 (107–2490)	46.7 (5.6–1412)	58.5 (5.6–189)
Alkaline phosphatase (39–117 unit/L)	449 (35–3384)	420 (35–3384)	190 (57–836)	267 (57–836)
